# The Meaning of Boarding in a Swedish Accident & Emergency Department: A Qualitative Study on Patients’ Experiences of Awaiting Admission

**DOI:** 10.3390/healthcare9010066

**Published:** 2021-01-12

**Authors:** Andreas Rantala, Sören Nordh, Mergime Dvorani, Anna Forsberg

**Affiliations:** 1 Department of Health Sciences, Lund University, SE-221 00 Lund, Sweden; anna.forsberg@med.lu.se; 2 Emergency Department, Helsingborg General Hospital, SE-251 87 Helsingborg, Sweden; soren.nordh@skane.se; 3 Centre of Interprofessional Cooperation within Emergency Care (CICE), Linnaeus University, SE-251 95 Växjö, Sweden; 4 Premedic AB, Ambulance Service Hässleholm, SE-281 25 Hässleholm, Sweden; mergime.dvorani@premedic.se; 5 Department of Cardiothoracic Surgery, Skåne University Hospital, SE-224 42 Lund, Sweden

**Keywords:** A&E, emergency department, suffering, qualitative study, phenomenological-hermeneutic, ethics, boarding, crowding

## Abstract

The number of in-hospital beds in Sweden has decreased during recent decades, resulting in the smallest number (2.2 available beds/1000 inhabitants) within the European Union. At the same time, the number of patients attending Accident and Emergency (A&E) departments has increased, resulting in overcrowding and boarding. The aim of this study was to explore the meaning of being subjected to boarding at an A&E department, as experienced by patients. A phenomenological-hermeneutic approach was chosen to interpret and understand the meaning of boarding at A&E. The study was carried out at a hospital in the south of Sweden. Seventeen participants with a mean age of 64 years (range: 35–86 years) were interviewed. The thematic structural analysis covers seven themes: Being in a state of uncertainty, Feeling abandoned, Fearing death, Enduring, Adjusting to the circumstances, Being a visitor in an unsafe place, and Acknowledging the staff, all illustrating that the participants were in a state of constant uncertainty and felt abandoned with no guidance or support from the clinicians. The conclusion is that the situation where patients are forced to wait in A&E, i.e., boarding, violates all conditions for professional ethics, presumably causing profound ethical stress in the healthcare professionals involved. Thus, boarding should be avoided.

## 1. Introduction

The number of in-hospital beds in Sweden has decreased during recent decades, resulting in the smallest number (2.2 beds/1000 inhabitants) within the European Union. In comparison, Germany provides eight beds per 1000 inhabitants, representing the highest ratio in Europe [1]. At the same time, the number of patients attending Accident and Emergency (A&E) departments has increased all over the world [2] as well as in Sweden [3], i.e., overcrowding, resulting in an increased length of stay at A&E [4]. Consequently, patients who need to be admitted are compelled to stay in A&E and experience the environment there while awaiting an in-patient bed at a hospital ward. The phenomenon of being admitted but not having a bed is known as boarding [5]. Hence, the rationale of this study is to explore in-depth the meaning of boarding and being subjected to one or more nights at A&E. 

Logistically, A&Es are characterised by three principal components as described by Asplin et al., [6] which influence the conditions there in different ways and facilitate understanding of the concept of overcrowding, i.e., input (e.g., patients’ chief complaints, patient flows and triage level or acuity), throughput (e.g., the number of clinicians, their workload and access to a treatment bed in A&E) and output (e.g., access to transport services and in-patient beds). As the patient inflow to A&E, i.e., the input component, increases and the access to in-patient beds is limited (i.e., the output), there is an obvious risk of patients having to board in A&E. Previous quantitative studies have revealed that boarding poses patient safety issues, including delays in receiving care, medical errors, adverse events and even death [7,8,9,10,11]. Little appears to have been done to find out what experience patients have had while waiting for an in-hospital bed, and thus this is the rationale of this study. However, what is known is that in cases of A&E boarding, longer hospital stays were expected, as well as potential threats to patient dignity [12,13]. 

Therefore, patients’ experiences of being a patient in an A&E setting should be considered. Patients emphasise the need for accurate and emphatic interpersonal communication, including active listening to their concerns while maintaining eye-contact and a calm tone of voice, which can alleviate anxiety [14,15]. This enables them to cope with their perceived illness and situation at A&E [16]. Relevant verbal information and explanations provided with clarity in a timely manner were appreciated and considered advantageous [17], making it possible for patients to endure the negative experience of long waiting times [15]. Leaflets and signs were clearly challenging, as patients reported problems with reading and retaining information [14,15]. Moreover, when information was lacking or unclear, e.g., when clinicians were vague about the diagnosis, it gave rise to uncertainty and could trigger anxiety [18,19]. Anxiety also seems to increase with the time spent at A&E due to worries about whether the delay is caused by the fact that a serious condition has been identified that requires further investigation, treatment or admission [20]. Generally, patients accepted the long wait and could understand that patients presenting with higher levels of acuity were prioritized [21]. In terms of the physical environment, A&E was perceived as unfamiliar and uncomfortable due to factors such as the absence of seclusion when waiting, noise and lighting [20]. In a study by Elmqvist et al., [22] patients felt that they were expected to be familiar with the unwritten rules set by the clinicians, which obstructed the initiation of a caring relationship. Furthermore, patients highlighted the importance of providing for basic physical needs, e.g., comfortable beds, toilets, food and drink [18]. Nurses who were responsive to patients’ fundamental bodily needs led to a positive experience [18]. Research on patients forced to stay at A&E due to a shortage of in-patient hospital beds is sparse. However, current evidence reveals that for patients, the meaning of boarding at A&E was waiting and hoping for a cure, described as being helpless and having no choice, an unavoidable challenge and mistrust of the healthcare system [23]. It has been found that many patients prefer boarding in a hallway of the hospital itself than in A&E [24,25,26]. 

Only a few studies of patients’ experiences of boarding at emergency departments have been published. Therefore, the aim of this study was to explore the meaning of being subjected to boarding at A&E, as experienced by patients. 

## 2. Materials and Methods 

A phenomenological-hermeneutic approach based on Ricoeur’s philosophy [27,28] was chosen to reveal the patients’ lived experiences, as well as to interpret and understand the meaning of boarding at A&E. The phenomenological-hermeneutic method developed by Lindseth and Norberg [29] was used to perform the analysis.

### 2.1. Setting and Participants 

This single centre study was carried out at the A&E department in one general county hospital in the south of Sweden. During 2018, the A&E in question handled some 75,200 visits, consisting of medical emergencies (for adults), orthopaedics, general surgery, infections and ear-nose-throat ailments. Unlike other countries, positions such as ramp nurses are generally not present in the Swedish A&E setting. Exclusion criteria were medically unstable, limited knowledge of the Swedish language and/or under the age of 18 years. During the study period, a total of 32 patients who experienced a decision to admit them to hospital, without a bed being available, were approached by an administrative nurse and invited to participate. Seventeen (nine women and eight men with a mean age of 64 years, range: 35–86 years) accepted. They received both written and oral information before providing their written informed consent. Their characteristics are presented in Table 1.

### 2.2. Data Collection

The interviews were performed from February to March 2019 within 14 days of discharge from the hospital. The interviews were conducted by two of the authors (S.N. and M.D.), of which the latter had no previous experience in an A&E context. The two interviewers are, like all authors, registered nurses. The participants chose a location for the interviews, all of which took place in the patient’s own home except for one, which was performed by phone. All interviews, which consisted of reflective and open-ended questions, were digitally recorded and transcribed verbatim shortly afterwards [30]. After a short recapitulation of the reasons for presenting at A&E, the interviews started with the question “Can you tell us about your recent visit to the A&E?”, and follow-up questions such as “Can you please describe…?” or “Can you please explain about…?” were posed to deepen, clarify and avoid misunderstanding. The mean duration of the interviews was 48.5 min (range: 28–72 min). 

### 2.3. Data Analysis

The first step, naïve reading consisted of reading each interview several times in order to grasp its meaning. The second step was the structural analysis, which aimed to capture the meaning of lived experience by identifying and formulating themes [29]. In this step, meaning units were identified, condensed, brought together and grouped into subthemes and themes (Table 2 and Table 3). The third and final step, comprehensive understanding [29], was performed by the authors reading the interview texts again and then reflecting together on the identified themes concerning the meaning of being subjected to boarding at A&E. The interpretation of the results was guided by the researchers’ pre-understanding from working in A&E (S.N.), nursing in an ambulance service context (A.R. and M.D.) or working in high tech hospital environments (A.F.), as well as the experience of caring for patients who had to stay overnight in A&E due to a lack of hospital beds (S.N.). 

### 2.4. Ethical Considerations

The ethical code of conduct was followed and we conformed to the ethical guidelines adopted by the Swedish Research Council. Consent, confidentiality, utility and information were taken into account in line with the Declaration of Helsinki [31] and Swedish ethical protocols and legislation (SFS 2003:460). Each participant received written and verbal information about the study. All participants gave their informed written consent and were assigned a number to ensure confidentiality. The study was approved by the Swedish Ethical Review Board in Lund (Dnr 2018/789). 

## 3. Results

The naïve understanding revealed that the participants were subjected to a state of limbo, admitted to a no man’s land of chaos and confusion. Therefore, the thematic structural analysis covers seven themes illustrating the meaning of being in limbo, i.e., a state of constant uncertainty and feeling abandoned with no guidance or support from clinicians, who were viewed with pity due to being totally stressed. While enduring the chaotic A&E environment filled with suffering fellow human beings, constant noise and unbearable lighting, the participants lost track of time while trying to cope and adjust without disturbing the staff. Being exposed and having no privacy increased the sense of loneliness and of being an abandoned visitor in a no man’s land, where they received no attention.

*You are put into no man’s land. You’re not supposed to be admitted to the A&E and because I’m not at any ward, nobody is responsible for me.*

(Informant 5)

### 3.1. Structural Analysis

An overview of the structural analysis is presented in Table 3. 

#### 3.1.1. Being in Uncertainty

Being in A&E and awaiting a hospital bed at a designated ward meant not receiving any information about when the transfer would take place. The uncertainty caused emotional distress, despair and the experience of being in a state of shock. They hoped for some form of information, even negative news, as knowing nothing was considered worse than knowing something. There was no one to talk to and the constant question: “how long am I supposed to wait?” was never answered, leading to a need to master the emotional stress and sense of hopelessness.

*Well, this is not good at all. It is a constant worry, what the fuck will happen now? And the constant question, how long will I be here and what will happen then? And no, there were no one I could talk to.*

(Informant 17)

#### 3.1.2. Feeling Abandoned

When the decision was made by the physician to admit the patient to in-hospital care, the participants felt neglected and abandoned on a stretcher in the corridor. They felt like an object and were subjected to the will and decision of the staff without any discussion or partnership.

*Everything was fine until I was transferred to the corridor, then I felt like… it’s difficult to find the words… I felt like an outcast, you are the fourth or fifth patient, sort of… we don’t care anymore.*

(Informant 2)

Nobody paid any attention unless the patients requested it, and if they were placed in the corridor, they did not receive a bell to summon for assistance, which led to a sense of helplessness.

*Actually, I was thinking, what if I have chest pain or fall from the stretcher, how will I manage? Of course, there are staff running around and they might care for me eventually, but it is very strange that I didn’t get a bell…*

(Informant 2)

When not being cared for, the only thing that remained was listening to the cries of fellow patients and waiting. As the hours passed, they lost track of time because nothing was happening.

*Nothing happens, you just lie there, thinking about what will happen, wondering if something will happen or not*

(Informant 14)

Being abandoned also meant being in discomfort and dealing with loneliness when offered nothing to eat or drink for eight hours or more. The discomfort was caused by being on a stretcher instead of in a proper hospital bed, waiting for hours for pain killers, not being looked after, and fearing that no one will hear you and that you are completely forgotten.

*Well, it was hugely distressing because I was lying there forever and I became more and more frustrated. The stretcher was awful and I had to ask for a proper bed. They gave me the bed and it was more comfortable, but then I had to wait and wait and nobody seemed to care. I heard the complaints of my fellow patients, how long are we supposed to wait? Aren’t you coming soon? …*

(Informant 17)

#### 3.1.3. Fearing Death

When left alone with illness and discomfort, there was nothing to distract from the fearsome thoughts of future mortality and it was easy to expect the worst. There was no one with whom to discuss the perception of one’s own situation, and the noisy environment, together with the moans of their fellow patients, created a wall of sound that increased the existential brooding.

*You simply believe that you might be suffering from all kinds of diseases. And how long will you live? Will someone come and tell you that in the best-case scenario you have seven months left to live? Or that I will lose my leg… Some people might be able to control their thoughts, but I certainly can’t.*

(Informant 1)

The loneliness also triggered an actual fear of dying in A&E without anyone noticing. They pondered on mortality, e.g., about being unconscious or not knowing the reason for their cardiac or abdominal symptoms.

*Will they try to save me if I have seizures? What happens if nobody notices me? The worst thing is the fear that I will lie here and die unnoticed.*

(Informant 15)

#### 3.1.4. Enduring

Lying on a stretcher in the corridor enabled a full view of the suffering of fellow patients who were in A&E at the same time. Hearing their cries or seeing their faces contorted in agony affected the participants both as human beings and as patients in a similar situation. Being confronted with the suffering of others made them depressed and sometimes increased their own suffering.

*Watching all these people coming here with various injuries and illnesses, is not a pleasant experience. You start feeling sorry for them.*

(Informant 1)

Being in the land of suffering evoked empathy with one’s fellow human beings, as well as a different perspective on one’s own illness and suffering. Comparing themselves with others sometimes relieved their own symptoms and made the whole situation more bearable.

*At the same time as you deal with your own suffering, you also suffer for them. And of course, they are there for a reason and obviously in great distress. But you can’t judge who is in the worst state.*

(Informant 5)

The A&E environment was filled with unpleasant noises and lights, making it impossible to rest.

*The environment could be far better, it is absolutely not good, neither for staff nor patients… somehow I got this feeling that I am in the waiting hall of a train station with people running everywhere…*

(Informant 1)

The lighting was perceived as terrible, and although the participants tried different ways of covering their heads or closing their eyes, it was impossible to shut out the light, even during the night. Being in the corridor also meant being exposed during tests and examinations or when unable to visit the toilet by oneself. While enduring the environment and complete lack of privacy, they were constantly aware of the suffering of others. As there was no privacy, they were forced to listen to other patients’ illness narratives and unintentionally became a part of their illness and everyday life. Thus, they not only had to endure their own illness and suffering, but also that of others.

*I overheard almost her entire life history which wasn’t nice at all. I certainly don’t need to know everything about her, a lot of sensitive stuff.*

(Informant 4)

#### 3.1.5. Adjusting to the Circumstances

A way of coping with the unpleasant situation was to justify one’s reasons for going to A&E in the first place. Another way of coping was to adopt a positive approach and appreciate even the slightest effort made by the staff to improve comfort.

*I certainly hoped to be transferred to a ward and when the decision was made that I would have to stay the night, they changed the stretcher to a proper bed. That was nice, not having to remain on that plastic stretcher and getting a real bed instead*

(Informant 11)

The participants tried to face reality and accept the current care conditions, where one important strategy was balancing expectations and being mentally prepared for a time-consuming process.

*The negative thing is that you were mentally prepared for having to wait forever because you read the newspapers*

(Informant 13)

Being dependent on others was perceived as difficult and demanding. Although the staff came when they rang the bell, it made them feel vulnerable.

*I found it difficult to have to use the bell for help. Of course the staff helped me that was not the problem. It simply made me feel more ill than I was.*

(Informant 3)

A way of mastering dependency and adjusting to the circumstances was trying not to care too much by simply accepting the environment and the situation, being patient and hoping for the best.

#### 3.1.6. Being a Visitor in an Unsafe Place

While waiting for a bed in the hospital ward, the participants feared that they would pick up an infection in the overcrowded A&E. They were also afraid that someone would steal their belongings as there were no lockers or secure areas where they could rest safely. Overall, the participants felt unsafe during their stay in A&E and expected the ward would be a safer place for treatment and recovery.

*I don’t think I would have made it for one more night because when she moved me out to the reception area I thought, “Oh my God”, there was someone with an infection right there beside me.*

(Informant 6)

#### 3.1.7. Acknowledging the Staff

In the land of suffering, the participants had plenty of time to observe the working conditions of the staff. They considered that their work environment was terrible, just as bad as the patients’ care environment, as they shared the same space, light and noise in addition to the constant stress. The participants genuinely had pity for the staff and expressed their empathy with them in all the interviews.

*When you have observed the absurd pressure, they’re under it must be crazy being a physician or a nurse at the A&E. Having to watch one stretcher after the other lined up along the walls of the corridors. They phone the hospital wards only to get the message that there are no beds available. Then, what to do? You simply do the best you can in an impossible situation.*

(Informant 13)

The participants tried their best to relieve the pressure on the staff by not disturbing them, making no demands and simply being as patient as possible and enduring the wait to be transferred to a safe place. 

## 4. Comprehensive Understanding and Discussion of the Findings

Our comprehensive understanding is that boarding at A&E means being in limbo and placed in the land of suffering, constituting an infringement of one’s freedom as a human being. As a patient boarding in A&E, one enters into a complete state of impotency due to the lack of security and privacy, where one is exposed and viewed as an object without any form of partnership or a caring relationship. Nothing makes sense and thus no sense of coherence is possible, despite the participants’ efforts to endure by imagining A&E as a space where “we are in this together, patients and professionals”. 

When interpreting the findings in the light of health geography and nursing geography, we focus on A&E not only as a space, but as a place that is both unsafe and uncaring. According to Kearns and Joseph [32], places hold particular significance for people, and a person’s background and experience may shape her/his impression of places and affect her/his opportunities and activities. By reading the newspaper or listening to hearsay, the participants were aware that A&E is a place characterized by waiting that offers neither opportunities nor activities but is instead experienced as the land of suffering, both others’ and one’s own. In nursing geography, it is argued that there is a dynamic between nursing, health and place [33]. As far back as the mid-19th century, Nightingale considered the importance of healthcare settings; their micro-environmental conditions such as ventilation, warmth and light, and their micro-social conditions such as nurses’ proximity to and intimate social interactions with their patients [33]. In A&E, the micro-environmental and the micro-social conditions limit comfort and proximity as well as intimate social interactions between nurses and patients. According to Piaschenko [34], a spatial perspective may be a useful way to think about the depth of the nurse-patient relationship due to the fact that the relationship itself is spatially determined. Peter [35] argues that place also has relevance to moral agency and activity by restricting or enhancing care and justice and affecting both personal and power relationships. Our understanding is that, as a place, A&E makes it impossible to create a safe space where a caregiver-patient relationship can be established, and it is also a place that restricts the patients’ autonomy and depowers them by preserving a state of uncertainty and abandonment. Caring has spatial features and relies on proximity, where physical proximity facilitates closeness when caregivers touch and physically act on behalf of patients. The conditions in A&E disconnect the caregiver from the patient, making it impossible for a narrative proximity to occur. Furthermore, moral proximity, which concerns acting and safeguarding patients’ interests, is also prevented by the uncaring space and place that constitutes A&E. Thus, the caregiver-patient spatial dynamic is ruined in A&E when caregivers are forced to distance themselves from the patients or engage in distal caring as described by Malone [36]. Keeping patients in A&E for several days due to a lack of in-hospital beds might be viewed as organized suffering caused by the hospital, and this undermines the solid foundation of healthcare, which is health promotion within a caring relationship. 

The meaning of places in healthcare delivery has gained little attention, as the location of services has been more important than what goes on inside. However, the experience of medicine or nursing cannot be detached from the place in which it is received, whether this be different types of setting such as hospitals, community clinics or homecare, or within those specific categories themselves [32]. A&E becomes an uncaring place offering nowhere to hide or rest, and as such the care provided is inhuman and profoundly unethical in the light of professional ethics. The period spent boarding in A&E becomes merely a waste of time [24] and energy consuming for the patient, causing profound moral distress for healthcare professionals [37] and a waste of financial resources for the hospital with no cost effectiveness [38]. Hospitals are about patient care and, to a great degree, care is characterized by nursing procedures and actions, which are influenced by health settings [33]. Nursing affects the meaning and function of a place and, in turn, a place changes the meaning and function of nursing. Thus, A&E as an insecure and extremely busy place changes the meaning and function of nursing. Patients typically remember nurses when recalling experiences of the healthcare they received and, in this way, nursing can be integral to the experiences associated with A&E. Allowing nurses to lead the change of the traditional patterns in A&E would presumably lead to improved micro-social conditions for both patients and professionals.

Being a professional nurse or physician in A&E involves an ethical demand to respond to the pledge to human beings, alive as well as deceased. Using Koehn’s [39] description of and argumentation about professional ethics, one could say that the unilateral, unqualified pledge of professionals to serve a specific group of vulnerable human beings, e.g., patients boarding in A&E, is the basis of professionals’ authority and legitimises their power to initiate and perform life-altering actions on the patient’s behalf. The pledge functions as a foundation, as it fulfils the objective requirements for a trusting relationship between the professional and the patient. It only binds the pledger and only legitimises the authority of those making the vow, as opposed to all human authority. In addition, the pledge can be said to be the foundation of professional authority because, like all foundations, it reveals in whose eyes professionals have authority. Those making the pledge have the authority to do what they promised to do, both in their own eyes and in those of their actual or potential patients. According to Koehn [39], adherence to the pledge fulfils the requirements for patients’ trust. The pledge itself can be considered as embodying these requirements. The origin of the structure does not affect its ability to serve as a legitimate foundation for professional practice. The question of legitimacy arises in every interaction with each patient, because in order to continue to merit a patient’s trust, the professional must repeatedly demonstrate that she/he is acting in that patient’s best interests, which is impossible in the A&E departments with overcrowded corridors. Koehn [39] lists seven conditions for professional authority. Applying these general conditions to the situation of boarding in A&E reveals the picture presented in Table 4. The situation when patients are forced to stay in A&E, i.e., boarding, violates all seven conditions of professional ethics, presumably causing profound ethical stress in the healthcare professionals involved, and as the patients are the ones who must pay the price, we argue that this situation should be avoided at all costs. 

Based on these findings, numerous implications might be relevant. Firstly, there is a need for establishing trustworthy caring relationships between caregivers and patients also in A&E. A designated nurse should be assigned to all patients staying in A&E for more than five hours. Secondly, relieving uncertainty should be a focus area during the patient’s whole stay since the pre-diagnostic phase is stressful for the patients. By providing “one-hour communication rounds”, the need for information and proximity might be satisfied. Thirdly, combining the regular communication rounds with patient safety aspects, i.e., checking the patients’ vitals and comfort needs, will act as a reminder that they are not neglected. Fourthly, healthcare professionals should apply temporary shielding walls as much as possible to create a sense of privacy despite being in a crowd. Finally, when rebuilding A&E, it is instrumental to consider the relational, spatial and ethical aspects of delivering care to facilitate caring relationships, including closeness and proximity between caregivers and patients.

### Methodological Considerations 

This is an attempt to explore the experiences of boarding in A&E in a European setting. One of the two authors who conducted the interviews has extensive prior knowledge of working at an A&E department. However, all other authors had only limited or no experience of the context in general or the phenomena, thus balancing pre-understanding and assumptions that could have a negative impact on the results. Due to the participants’ illness conditions in combination with the fact that it was not possible to offer a secluded place, the interviews were conducted post discharge. Thus, recall bias cannot be ruled out and must be taken into consideration when interpreting the findings. The participants in this study were evenly distributed between men and women and the median age was 64 years. While there is a risk that younger patients’ experiences are missing, most patients attending A&E belong to the older part of the population. The main limitation is that the sample only included Swedish speaking participants of Swedish origin, and thus it fails to reflect the ethnic diversity that is increasingly becoming a reality in Swedish healthcare. In addition, the study was conducted at a single centre. Thus, it cannot be ruled out that a study performed at another hospital could have a somewhat different result. As patients are present at A&E due to illness, there were patients with disorders that, e.g., involved fatigue or infectious diseases who were not approached.

## 5. Conclusions

The meaning of boarding at A&E is being in a state of limbo and placed in a land of suffering, possibly constituting an infringement of one’s freedom as a human being. The situation where patients are forced to stay in A&E, i.e., boarded, possibly violates all seven conditions of professional ethics.

## Figures and Tables

**Table 1:** Patient characteristics (*n* = 17).

Characteristics	
Male	8 (47%)
Female	9 (53%)
Median age (range)	64 (35–86)
Walk-in admissions	14 (82%)
Transferred to ward	11 (65%)
Average length of stay (range)	22 h 22 m (16 h 21 m–28 h 23 m)
Average stay as boarded (range)	18 h 10 m (8 h 27 m–22 h 55 m)

Average length of stay—the total time at A&E, while Average stay as boarded – the time from the decision to admit to transfer to a hospital ward.

**Table 2:** Example of the structural analysis.

Meaning Unit	Condensation	Sub-Theme	Theme
“And then nobody cared that I was lying there, whimpering and that I gasped for air”.	Nobody cared	Feeling neglected	Feeling abandoned
“Well, it was hugely distressing because I was lying there forever, and I became more and more frustrated. The stretcher was awful, and I had to ask for a proper bed. They gave me the bed and it was more comfortable, but then I had to wait and wait, and nobody seemed to care. I heard the complaints of my fellow patients, how long are we supposed to wait? Aren’t you coming soon?”	Being in distress on an awful stretcher	Being in discomfort	Feeling abandoned

**Table 3:** Structural analysis of being in limbo among 17 patients.

Sub-Themes	Themes
Lacking information and piloting Mastering emotional stress Feeling hopeless	Being in a state of uncertainty
Feeling neglected Losing track of time Being an object Being in discomfort Dealing with loneliness Feeling helpless	Feeling abandoned
Expecting the worst Pondering on mortality	Fearing death
Being constantly aware of others’ suffering Feeling empathy with other patients Gaining perspectives Having no rest due to constant noise Feeling exposed	Enduring
Justifying reasons for seeking care at A&E Adopting a positive approach Accepting facts Balancing expectations Mastering dependency Trying not to care	Adjusting to the circumstances
Fear of being infected Protecting one’s belongings Feeling insecure Having no privacy	Being a visitor in an unsafe place
Feeling empathy with the staff Having pity for the staff Making no demands	Acknowledging the staff

**Table 4:** The seven conditions for professional authority described by Koehn.

Condition	Discussion Related to the Findings of the Study
1. To be trustworthy, staff must have the patient’s interest at heart, including that of her/his relatives.	While boarding, several patients were exposed to the physical environment of A&E, restricting their opportunity to rest and recuperate. By, e.g., providing temporary shielding walls, patients’ trust could be maintained.
2. The best evidence of professional staff doing their utmost for the patient’s good is acting on behalf of the patient and her/his relatives.	Demonstrating willingness to act is necessary for trust in this situation. The participants in the present study felt empathy with the staff members’ situation, as they realised and acknowledged that the staff had a difficult and stressful job in poor conditions. Thus, the patients accepted that the staff did as well as they could in the prevailing situation.
3. The willingness must be sustained, as the patient expects the professional’s good will to be forthcoming, not only for the next minute or hour, but for as long as it takes.	Patients experiencing boarding expect the willingness to last until they are transferred to a hospital ward.
4. Even sustained willingness to help will not make a professional trustworthy unless she/he is competent in terms of treating the patient’s condition and does what will in fact help the patient.	Patients presenting at A&E have been shown to suffer when struggling to take control of the situation and when treated like objects [40]. In short, to be trustworthy, professionals must be competent.
5. The professional must be able to demand that the patient exhibits the degree of accountability and discipline necessary for treatment to proceed.	When A&E is crowded and patients are boarded, the staff are forced to deal with urgent patients, as well as boarded patients. Hereby, patients are compelled to take responsibility for their own situation, whether or not they are able.
6. The trustworthy professional must have the freedom to act in the best interest of each patient.	Revising prior commitments and previous allocations of time and energy might result in a better service for the patient. The participants found that the staff acted in the best interest of each patient. However, knowing a person’s needs but being unable to provide the necessary care is stressful for staff and could possibly result in compassion fatigue and burnout [41].
7. The professional must have a highly internalized sense of responsibility. No one can supervise professionals all the time, so the professional her/himself must monitor her/his own behaviour.	The A&E staff must critically evaluate their behaviour and their efforts to relieve the suffering caused by boarding and their role in this way of organizing care.

Conditions for professional authority described by (Koehn [39] (pp. 54–56)).

## Data Availability

The data presented in this study are available on reasonable request from the corresponding author. The data are not publicly available due to the confidential nature of participants transcript data.
